# The Biomechanical Effects of Kinesiology Taping Methods on Side-Step Cutting Movements in Chronic Ankle Instability

**DOI:** 10.3390/healthcare12242561

**Published:** 2024-12-19

**Authors:** Xuting Wang, Wenjing Quan, Yiwen Ma, Sarosi Jozsef, Yufei Fang, Yaodong Gu

**Affiliations:** 1 Faculty of Sports Science, Ningbo University, Ningbo 315211, China; 2311040088@nbu.edu.cn (X.W.); 2311040015@nbu.edu.cn (Y.M.); 2 Faculty of Engineering, University of Szeged, 6720 Szeged, Hungary; sarosi@mk.u-szeged.hu; 3 Department of Radiology, Ningbo No. 2 Hospital, Ningbo 315010, China; fyf369@126.com

**Keywords:** kinesiology taping methods, side-step cutting movements, chronic ankle instability

## Abstract

**Background**: The ankle joint is among the most vulnerable areas for injuries during daily activities and sports. This study focuses on individuals with chronic ankle instability (CAI), comparing the biomechanical characteristics of the lower limb during side-step cutting under various conditions. The aim is to analyze the impact of kinesiology tape (KT) length on the biomechanical properties of the lower limb during side-step cutting, thereby providing theoretical support and practical guidance for protective measures against lower-limb sports injuries. **Methods**: Twelve subjects with CAI who met the experimental criteria were recruited. Each subject underwent testing without taping (NT), with short kinesiology tape (ST), and with long kinesiology tape (LT), while performing a 45° side-step cutting task. This study employed the VICON three-dimensional motion capture system alongside the Kistler force plate to synchronously gather kinematic and kinetic data during the side-step cutting. Visual 3D software (V6.0, C-Motion, Germantown, MD, USA) was utilized to compute the kinematic and kinetic data, while OpenSim 4.4 software (Stanford University, Stanford, CA, USA) calculated joint forces. A one-way Analysis of Variance (ANOVA) was conducted using SnPM, with the significance threshold established at *p* < 0.05. The Origin software 2021 was used for data graphic processing. **Results**: KT was found to significantly affect joint angles, angular velocities, and moments in the sagittal, frontal, and transverse planes. LT increased hip and knee flexion angles as well as angular velocity, while ST resulted in reduced ankle inversion and increased knee internal rotation. Both types of KT enhanced hip abduction moment and knee adduction/abduction moment. Additionally, LT reduced the ankle joint reaction force. **Conclusions**: These findings suggest that the application of KT over a short duration leads to improvements in the lower-limb performance during side-step cutting motions in individuals with CAI, thus potentially decreasing the risk of injury.

## 1. Introduction

Lateral ankle sprain (LAS) is a prevalent sports injury, notable for its high recurrence rate among lower-limb musculoskeletal injuries [1]. Following an ankle sprain, approximately 40% of individuals develop ankle instability, which often leads to chronic ankle instability (CAI) [2,3]. CAI is characterized by a minimum of two significant ankle sprains and/or recurrent sprains of the affected ankle within the past six months, as well as a sensation of instability or weakness in the ankle during daily activities [4]. Furthermore, CAI has been identified as a potential contributor to a range of sports-related disorders [5]. Therefore, it is imperative to implement proactive measures to avert the transition from LAS to CAI.

Patients with CAI exhibit altered lower-limb biomechanical characteristics, including increased ankle plantar flexion, larger inversion angles, and heightened inversion angular velocity compared to healthy individuals [6]. Previous studies have shown that individuals with CAI display modified kinematic patterns during movements that affect the hip, knee, and ankle joints [7,8,9]. The side-cutting maneuver, characterized by abrupt stops and changes in direction, significantly alters the biomechanical demands placed on lower-limb joints, increasing multi-planar loading on both the knee and ankle joints, which could elevate the risk of injury [10,11]. During side-cutting movements, individuals with CAI show a decreased ankle eversion moment as well as knee abduction moment, along with an increased ankle plantar flexion moment, in comparison to healthy participants [12,13]. Concurrently, those with CAI may demonstrate altered kinematics in proximal joints, including increased angles of hip and knee flexion, along with greater hip abduction angles. Studies have also shown that participants with CAI display greater hip and knee flexion compared to the control group during the stance phase, along with increased hip abduction both prior to initial contact and throughout the stance phase [6,14]. Koshino et al. found that individuals with CAI exhibited increased hip flexion and greater ankle inversion during side-cutting movement compared to the healthy individuals [15]. These altered ankle kinematics may clarify their susceptibility to ankle instability or lateral sprains during the side-cutting motion.

Kinesiology tape (KT) is an adhesive tape designed to stretch to approximately 90% to 140%, applying continuous traction and shear forces on the skin, which distinguishes it from traditional athletic tape [16]. Research has demonstrated its effectiveness in lowering the occurrence of lateral ankle sprains in individuals with a prior history of these injuries [17]. In recent years, KT has gained popularity for its application in preventing and rehabilitating sports injuries. Zhang et al. found that KT decreases the knee eversion angle in athletes who have had anterior cruciate ligament reconstruction during cutting maneuvers, potentially lowering the risk of secondary injuries [18]. Additionally, Jo et al. examined the impact of KT on lower-limb biomechanics in patients with CAI during cutting movements. Their results indicated that the application of KT decreased ankle inversion, as well as the ranges of motion for knee flexion and hip flexion and internal rotation, while simultaneously enhancing ankle stability [19]. Several studies have demonstrated that the use of KT significantly reduces the plantar flexion angle of the ankle in patients with CAI during a drop task [20,21]. Furthermore, recent evidence highlighted in a review indicates that KT could decrease both the plantar flexion and inversion angles of the ankle during the swing phase of walking in CAI patients [22]. Additionally, Cheraghi et al. reported that KT significantly increased the range of motion in the ankle joint plantar flexion while reducing the range of motion in the coronal plane for patients with CAI [23]. Moreover, some research findings indicate that the KT group exhibits significantly reduced peak sagittal plane ground reaction force (GRF), ankle joint reaction force, and peak ankle plantarflexion moment during landing tasks [6,24]. Moore et al. conducted a study on the application of tape in healthy males during side-cutting movements, analyzing the kinematic and kinetic factors of the hip, knee, and ankle joints. The findings indicated that the application of KT did not significantly alter movement patterns or mechanical performance. This outcome may be influenced by factors such as the taping technique, subject selection, and recruitment processes [25]. Furthermore, studies suggest that KT can enhance ankle inversion proprioception during landing in both individuals with and without CAI. Yu et al. demonstrated that KT effectively improves ankle proprioception [26]. Additionally, Chunapis et al. suggested that KT could enhance both ankle proprioception and static balance in individuals with CAI [27].

Previous research has demonstrated that CAI does not impact contact forces at proximal joints [28]. Joint contact forces result from the combined effects of GRF and muscle forces [29,30]. Kim et al. demonstrated that during side-step cutting, individuals with CAI exhibit greater ankle joint forces compared to healthy individuals [31]. Nevertheless, the effects of KT on joint forces in individuals with CAI remain unclear. Recent research has increasingly focused on the effects of varying lengths of KT on CAI. Yu et al. suggest that mid-to-long-length taping can effectively enhance ankle proprioception [26]. Jackson demonstrated that the prolonged application of KT to the ankle for 48 h can improve balance compared to baseline measurements, particularly among individuals with CAI who did not receive KT application [32]. Theoretically, a longer length of KT may cover a larger area of skin, thereby providing increased proprioceptive input, which could enhance athletic performance [26,32]. However, few studies have investigated the effects of KT length on lower-limb biomechanical parameters during side-cutting movements. Furthermore, the impact of cross-joint taping—linking the ankle joint with its adjacent knee joint through taping—on the biomechanics of the CAI ankle and other lower-limb functions remains unclear [26].

The purpose of this study was to explore the impact of different lengths of KT on the kinematics and kinetics of the lower limb during side-step cutting in individuals with CAI. We hypothesize that varying lengths of KT application will induce distinct biomechanical changes at the hip, knee, and ankle joints during side-step cutting in individuals with CAI. Specifically, this study expects that LT application will result in increased hip and knee flexion angles and angular velocities, whereas ST application is anticipated to reduce ankle inversion and increase knee internal rotation.

## 2. Materials and Methods

### 2.1. Participants

Twelve subjects (age: 23.85 ± 0.79 years; height: 180.7 ± 8.75 cm; weight: 79.85 ± 8.80 kg; CAIT scores: 16.71 ± 2.98) with CAI were recruited for this study through completion of the Cumberland Ankle Instability Tool (CAIT) questionnaire. The selection criteria for CAI subjects were as follows: inclusion criteria: (1) The patient had experienced at least one ankle sprain accompanied by subsequent pain and swelling [33]. (2) The patient reported episodes of perceived ankle instability without recurrent sprains within the past six months. (3) The Cumberland Ankle Instability Tool (CAIT) score for the affected side was ≤24 points [34]. (4) The subject maintained a consistent daily activity level throughout the study period. Exclusion criteria: (1) The presence of other severe diseases, injuries, sensory disorders, etc. (2) The acute phase of an ankle sprain. (3) Severe skin conditions or allergies to taping materials. The research protocol was approved by the Ethics Committee of Ningbo University Research Institute (RAIGH202407011185), and all participants provided informed consent.

### 2.2. Experimental Procedure

**Figure 1:**
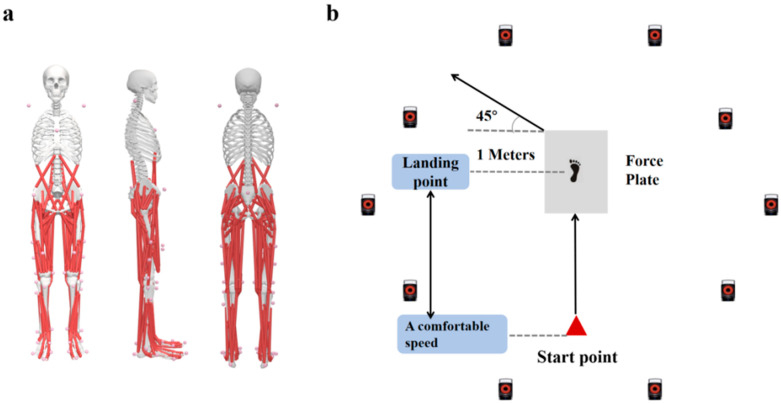
(**a**) Reflective marker’s front, side, and back position on subjects. (**b**) 45° side-step cutting experiment workflow.

This study utilized the Vicon infrared motion capture system, equipped with 10 infrared cameras (Vicon Metrics Ltd., 10 MX-T20 cameras, Oxford, UK), to collect three-dimensional kinematic data during side-step cutting at a sampling frequency of 200 Hz. Simultaneously, a Kistler force plate (Advanced Mechanical Technology, Inc., Watertown, MA, USA) was employed to collect the ground reaction force with a sampling frequency of 1000 Hz. All subjects were asked, in uniform tight-fitting clothing and barefoot, to ensure that the body markers used for motion capture were visible. Their anthropometric measurements, including height, weight, and leg length were recorded. Before the experiment, participants completed a 5 min warm-up on the treadmill. According to the Opensim Gait-2392 model, 38 markers (14 mm) were affixed to the lower limb and torso [35] (Figure 1a). Randomly conducted taping or barefoot collection on participants, which included NT, ST, and LT. A 1 m-wide path was set at the 45° side-cut position, and participants were instructed to step into the path with their non-supporting foot [15] (Figure 1b). Before the commencement of the experiment, a static calibration procedure was completed to facilitate the subsequent establishment of a human model.

Each participant completed four side-step cutting movements under each taping condition. The subjects perceived the running speed as comfortable [13]. The movement requirements were as follows: upon reaching the force plate, the participant positioned their right foot on the force plate as well as stepped 45° to the left with their left foot into the 1 m-wide path to complete the turn, thereby performing the 45° side-step cutting movement. If the marker points fell off or the foot did not fully contact the force platform during the experiment, the trial was repeated. Ultimately, four successful data collections were recorded.

### 2.3. Taping Procedure

**Figure 2:**
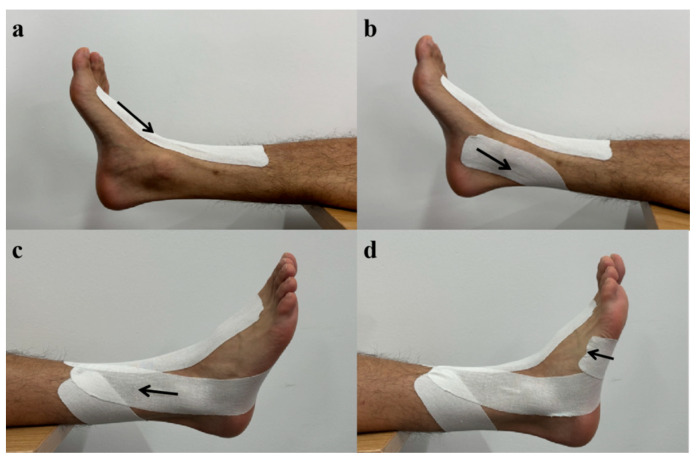
The short tape technique. (**a**) The first tape. (**b**) The second tape. (**c**) The third tape. (**d**) The fourth tape.

**Figure 3:**
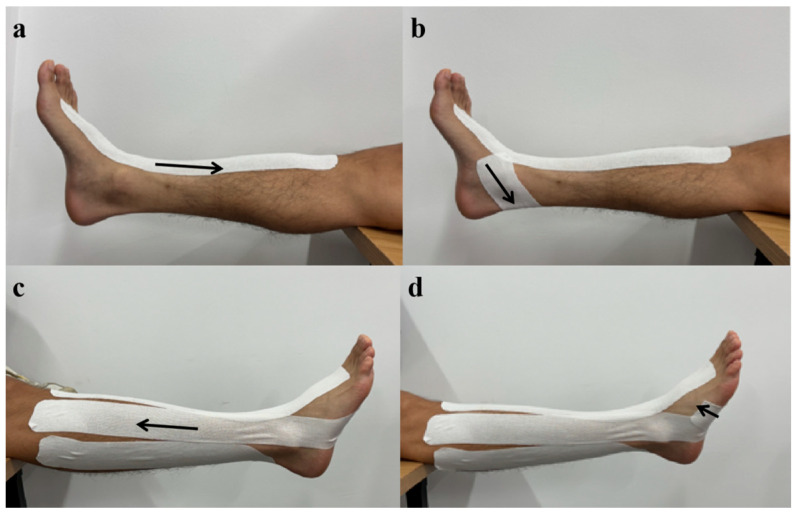
The long tape technique. (**a**) The first tape. (**b**) The second tape. (**c**) The third tape. (**d**) The fourth tape.

In this study, KT with a width of 5 cm was utilized, which is a commercially available product, and its maximum stretch length is approximately 90% to 140% [16]. The application technique for the KT followed the methodology described by Jackson et al. [32]. The current study systematically varied the length of the taping, incorporating both short lengths, which involved only the foot and ankle complex (Figure 2), and long lengths that extended below the knee [33] (Figure 3). The short tape technique employed four distinct strips: (1) The first tape anchor point was located at the first cuneiform and first metatarsal on the dorsum of the foot, extending upward in the direction of the anterior tibial muscle and terminating 6 cm above the ankle (Figure 2a). (2) The second tape anchor point was positioned at the first metatarsal on the plantar surface, wrapping around the lateral malleolus outward, extending in the direction of the peroneus longus muscle, and concluding 6 cm above the ankle (Figure 2b). (3) The third tape anchor point was located at the medial malleolus, extending backward along the posterior medial edges of the tibia and fibula, and ending 6 cm above the ankle (Figure 2c). (4) The fourth tape anchor point was situated on the anterior side of the lateral malleolus, extending to the plantar surface and concluding at the transverse arch (Figure 2d). The long tape technique involved the use of four distinct strips: (1) The first tape anchor point is located at the first cuneiform and first metatarsal on the dorsum of the foot, extending upward in the direction of the anterior tibial muscle and terminating at the tibial plateau (Figure 3a). (2) The second tape anchor point is situated at the first metatarsal on the plantar surface, wrapping around the lateral malleolus outward, extending toward the peroneus longus muscle, and concluding at the fibular head (Figure 3b). (3) The third tape anchor point is positioned at the medial malleolus, extending backward along the posterior medial edges of the tibia and fibula, and ending below the popliteal fossa (Figure 3c). (4) The fourth tape anchor point is located on the anterior side of the lateral malleolus, extending to the plantar surface and terminating at the transverse arch (Figure 3d). A moderate level of tension, ranging from 20% to 35%, was applied when each piece of tape was affixed to the participant’s skin and was removed immediately after data collection [26,32]. To ensure consistent KT tension applied to the ankle and knee joints, the length of the ankle was taken into account when calculating the KT length using Equation (1) [36].

Taping cutting length= [(actual length − 8 cm/1.5) + 8 cm] × 1.10(1)

### 2.4. Data Processing

**Figure 4:**
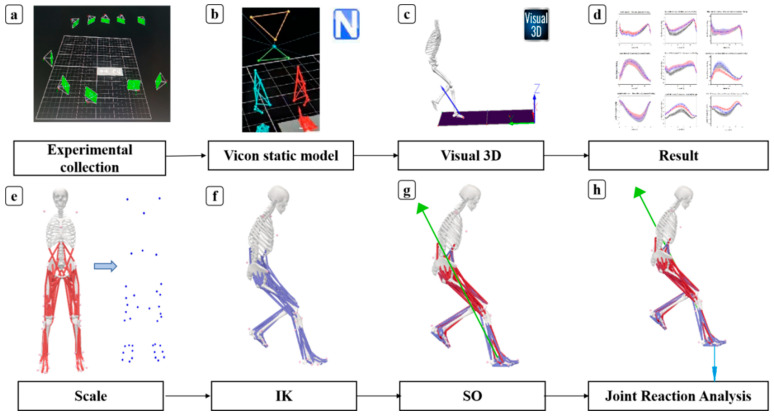
Workflow of data processing. (**a**) Experimental collection. (**b**) Static model. (**c**) The data processing of Visual 3D. (**d**) The results of hip, knee, and ankle joint moments. (**e**) Scaling of the model. (**f**) Inverse kinematics (IK). (**g**) Static optimization (SO). (**h**) Joint reaction analysis.

Data processing for both the static and cutting motion of participants was conducted using the Vicon Nexus 2.9.1 software (200 Hz, Oxford Metrics, Oxford, UK) (Figure 4a). Initially, markers were linked according to the selected lower-limb model, and missing markers in static and side-step cutting actions were interpolated (Figure 4b). This study specifically focused on the support phase of the cutting motion, defined as the period starting from when the right leg first made contact with the ground (vGRF > 20 N) until the moment it lifted off (vGRF < 20 N) [37]. Subsequently, the data were processed using Visual 3D (V6.0, C-Motion, Germantown, MD, USA), with a low-pass Butterworth digital filter applied at 20 Hz for kinetic data and 10 Hz for kinematic data [38] (Figure 4c). A Cardan X-Y-Z rotation sequence was used to determine the angles of the ankle, knee, and hip joints. The moments at the lower-limb joints were calculated using inverse dynamics based on the Newton–Euler method [39] (Figure 4d). The dynamic parameters were further standardized based on each subject’s body height and weight. Each dataset was normalized to 101 data points, corresponding to the frames within the standing phase.

The data were exported in C3D format using a customized MATLAB R2023a (The Math Works, Natick, MA, USA) script, resulting in the generation of “trc” and “mot” files. Opensim 4.4 (Stanford University, Stanford, CA, USA) was employed to calculate the joint reaction forces in the lower limb [40]. The detailed steps are as follows: The model was subjected to scaling calibration to achieve anthropometrically accurate representations for each subject, ensuring that muscle origins and insertions, and muscle moment arms, were consistent with the subject’s limb lengths (Figure 4e). Joint angles were computed using an Inverse Kinematics (IK) algorithm, which effectively minimized the discrepancy between virtual points in the model and experimental markers recorded during the study (Figure 4f). Joint moment was calculated using an Inverse Dynamics (ID) algorithm, providing insights into the biomechanical forces acting at the joints. Static optimization (SO) algorithms were employed to estimate muscle activations and forces, offering a detailed understanding of the muscular contributions to joint dynamics (Figure 4g). Joint reaction forces at the hip, knee, and ankle were computed using an analytical tool that incorporated a joint reaction algorithm, elucidating the mechanical interactions within these critical joints [41,42] (Figure 4h).

### 2.5. Statistical Analysis

This study utilized statistical nonparametric mapping (SnPM) to perform a one-way Analysis of Variance (ANOVA) [43,44] involving three independent groups, the NT, the ST, and the LT groups. The variables included joint angles, joint angular velocities, and joint moments, as well as joint reaction force during the contact phase of the side-step cutting action. Within the SnPM framework, a one-way ANOVA was conducted, followed by paired sample t-tests for post hoc analyses. The alpha risk for the post hoc tests was adjusted using the Bonferroni correction. The analysis was performed in MATLAB R2023a with a significance level of 0.05.

## 3. Results

All statistical findings are presented below. The supplementary materials include the figures that show no significant differences.

### 3.1. Kinematics

**Figure 5:**
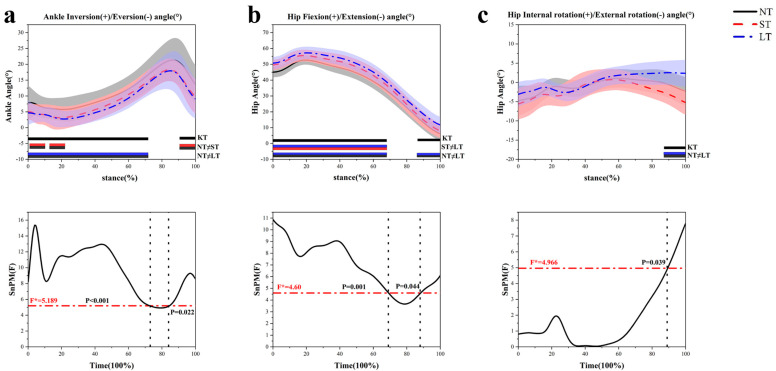

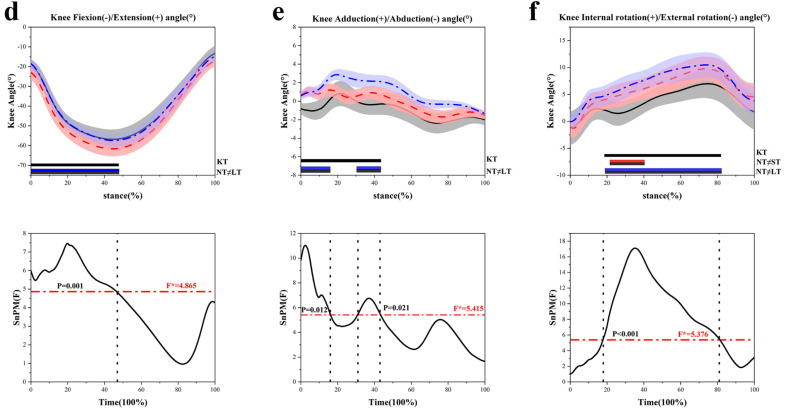
The kinematic characteristics of the lower-limb joints during the side-step cutting stance phase. (**a**) The ankle joint in the frontal plane. (**b**) The hip joint in the sagittal plane. (**c**) The hip joint in the horizontal plane. (**d**) The knee joint in the sagittal plane. (**e**) The knee joint in the frontal plane. (**f**) The knee joint in the horizontal plane.

In comparison to barefoot conditions, the use of LT significantly increased the hip flexion angle at 0–69% (*p* = 0.001) and 88–100% (*p* = 0.044) (Figure 5b). Additionally, both hip joint flexion angles at 0–69% (*p* = 0.001) and knee joint flexion angles at 0–47% (*p* = 0.001) (Figure 5d) were greater with LT than with ST, while no significant difference was observed in the ankle joint angle. ST resulted in a reduction in the inversion angle of the ankle joint at 1–9% (*p* < 0.001), 12–23% (*p* < 0.001), and 90–100% (*p* = 0.022) compared to NT (Figure 5a). LT also demonstrated a smaller inversion angle of the ankle joint at 0–72% (*p* < 0.001) and a significantly greater adduction angle of the knee joint at 0–16% (*p* = 0.012) and 31–43% (*p* = 0.021) during initial contact relative to ST conditions, with no notable differences in the hip joint (Figure 5e). In the transverse plane, both ST and LT increased the knee internal rotation angle at 24–40% (*p* < 0.001) and 18–81% (*p* < 0.001) compared to NT (Figure 5f). Furthermore, compared to ST, LT increased the hip external rotation angle at 89–100% (*p* = 0.039) (Figure 5c), with no significant difference in the ankle joint angle.

**Figure 6:**
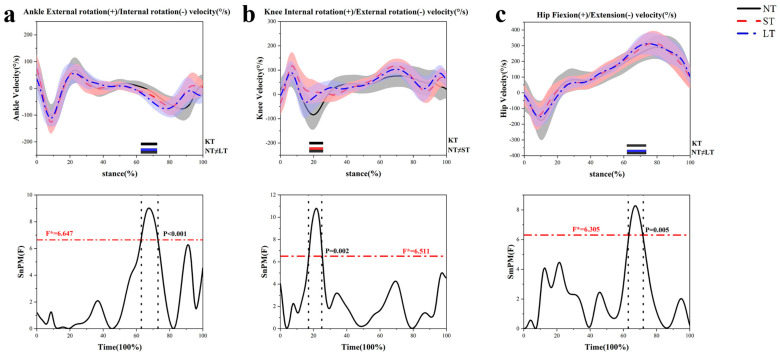

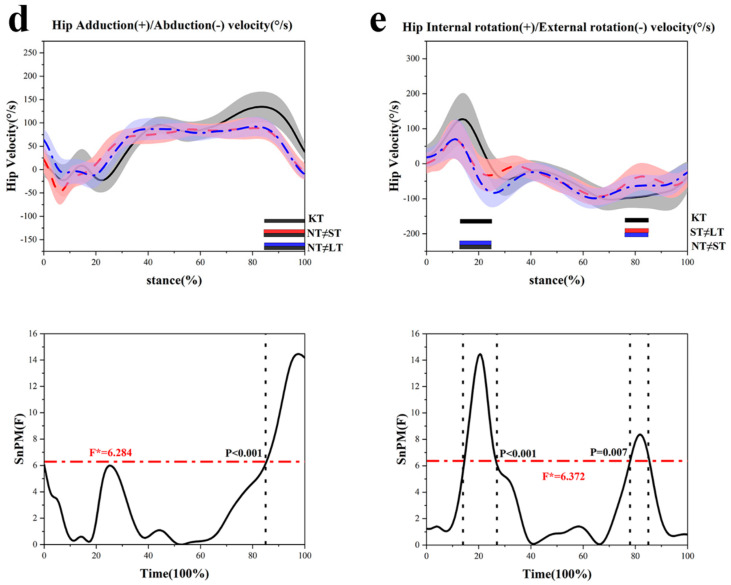
The lower-limb joints’ velocity in the sagittal, frontal, and transverse planes during the stance phase in side-step cutting. (**a**) The ankle joint in the horizontal plane. (**b**) The knee joint in the horizontal plane. (**c**) The hip joint in the sagittal plane. (**d**) The hip joint in the frontal plane. (**e**) The hip joint in the horizontal plane.

In terms of hip joint velocity, LT exhibited a greater angular velocity of hip flexion at 63–72% (*p* = 0.005) when compared to ST (Figure 6c). In the frontal plane, both ST and LT showed a reduction in hip adduction angular velocity at 85–100% (*p* < 0.001) relative to NT (Figure 6d). In the transverse plane, ST demonstrated an increase in the internal rotation angular velocity of the knee at 17–25% (*p* = 0.002) (Figure 6b), while LT showed a decrease in the internal rotation angular velocities of both the hip at 14–27% (*p* < 0.001) (Figure 6e) and the ankle at 63–73% (*p* < 0.001) (Figure 6a). Conversely, ST revealed an increase in hip external rotation angular velocity at 78–85% (*p* = 0.007) in comparison to LT (Figure 6e).

### 3.2. Kinetics

**Figure 7:**
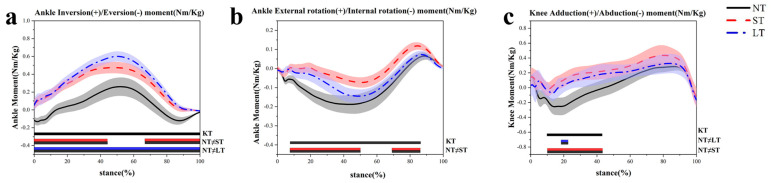

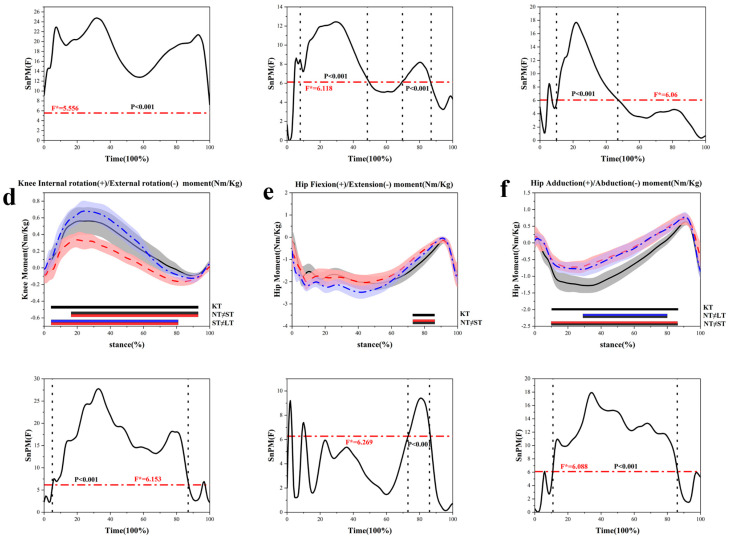
The kinetics characteristics of the lower-limb moments during the side-step cutting stance phase. (**a**) The ankle joint in the frontal plane. (**b**) The ankle joint in the horizontal plane. (**c**) The knee joint in the frontal plane. (**d**) The knee joint in the horizontal plane. (**e**) The hip joint in the sagittal plane. (**f**) The hip joint in the frontal plane.

During the landing phase, the hip flexion moment increased by 73–86% (*p* < 0.001) when using the ST condition compared to NT (Figure 7e). Both ST and LT conditions resulted in increases in the hip abduction moment of 11–86% and 29–80%, respectively (*p* < 0.001) (Figure 7f). Increases were also found in the adduction/abduction moments of the knee, with changes of 10–47% and 18–26% (*p* < 0.001) (Figure 7c), as well as in the inversion/eversion moments of the ankle, which ranged from 0 to 100% (*p* < 0.001) (Figure 7a) compared to NT. The use of ST increased the internal rotation moment of the ankle at 8–50% and 69–87% (*p* < 0.001) (Figure 7b) while simultaneously reducing the internal rotation moment of the knee by 16–87% (*p* < 0.001) compared to NT (Figure 7d). In contrast, LT increased the internal rotation moment of the knee by 5–81% (*p* < 0.001) compared to ST (Figure 7d), with no notable changes observed in the hip joint.

**Figure 8:**
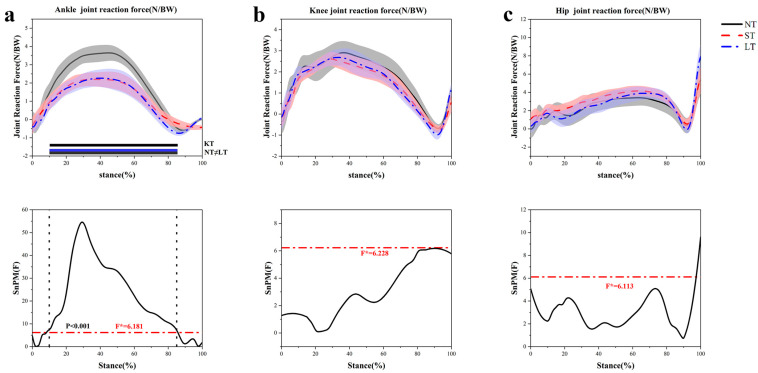
Joint reaction force characteristics during the support phase. (**a**) Hip joint reaction force. (**b**) Knee joint reaction force. (**c**) Ankle joint reaction force.

The ankle joint reaction force decreased at 10–85% (*p* < 0.001) with LT compared to NT (Figure 8a), with no significant difference observed in the hip (Figure 8c) and knee joint (Figure 8b).

## 4. Discussion

This study utilized an intervention approach involving the application of KT in different lengths on the lower limbs to investigate its effects on the kinematic and kinetic parameters of the hip, knee, and ankle joints in three-dimensional space during side-step cutting in patients with CAI. Consistent with the hypothesis, the findings revealed that KT application may alter the kinematic and kinetic parameters of CAI in the short term. LT application increased hip and knee flexion angles and angular velocity, while ST application resulted in reduced ankle inversion and increased knee internal rotation. Both types of KT application led to an increase in the hip abduction moment and knee adduction/abduction moments. Furthermore, LT application also reduced the ankle joint reaction force. Additionally, a comparison of the biomechanical data indicated that the improvement effect of LT was more pronounced than that of ST.

The angle of hip flexion is recognized as a significant factor in predicting the occurrence of injuries. In this study, the hip flexion angle under KT conditions showed a tendency to increase compared to NT. Research on the injury mechanisms of subjects suggests that an increase in the hip flexion angle at the initial foot contact enhances the hip joint’s buffering capacity, thereby reducing the impact transmitted to the knee joint [45]. Active hip flexion contributes to an increase in the knee flexion angle, which helps to mitigate the load exerted on the knee by GRF. LT resulted in greater hip and knee flexion angles compared to ST, suggesting that longer tapes may provide better buffering effects during the landing phase of CAI without compromising joint mobility and athletic performance. A smaller knee flexion angle at initial foot contact is regarded as a potential indicator of higher injury risk [46]. Studies by David et al. highlight that a knee flexion angle of less than 30° at the initial contact during side-step cutting movements is a high-risk factor for injury [47]. Other research indicates that a knee flexion angle below 30° significantly increases the tensile load on the anterior cruciate ligament, leading to an increase in anterior tibial shear force, thereby elevating the risk of injury [48]. Consequently, an excessively small knee flexion angle in the sagittal plane is also regarded as a major risk factor for knee injuries. In this study, the knee flexion angle at initial contact significantly increased with KT compared to NT, suggesting that taping may effectively enhance knee flexion angles during side-step cutting. Excessive valgus knee motion has been widely recognized by numerous scholars as a significant mechanism for anterior cruciate ligament (ACL) injury [49]. Researchers such as Donelon [11] conducted a systematic review of the literature on lateral cutting movements, revealing that an excessively large eversion angle at the initial contact phase of landing is a crucial risk factor for injury occurrence. Compared to NT, the use of KT reduced the eversion angle of the knee, aligning with findings from other studies. Therefore, optimizing the eversion angle of the knee through KT is of significant importance for preventing injuries and maintaining knee health. The ankle is recognized as a commonly affected anatomical area [50]. A larger ankle plantar flexion angle at landing during side-step cutting is considered a risk factor for ankle sprains. The anatomical structure of the talus, which is wider anteriorly and narrower posteriorly, creates a gap between the posterior aspects of the talus and the ankle mortise during plantar flexion. This configuration results in a relatively unstable state and an increased risk of sprain [51]. Wright et al. [52] found that a greater ankle plantar flexion angle correlates with a higher incidence of ankle sprains, as demonstrated using an ankle sprain model. However, research on ankle taping has produced varied results. Liu et al. observed that while KT could increase knee flexion angles, it had minimal effect on ankle angles during stop–jump tasks. In our study, we found no significant difference in ankle plantar flexion angles between KT and NT conditions, possibly due to differences in taping techniques and application locations. Migel et al. [22] reviewed that KT could reduce ankle plantar flexion and inversion angles during the swing phase of walking in patients with CAI, indicating that KT positions the ankle in a state less prone to sprains, thus lowering the risk of injury. This study demonstrated that KT significantly reduced ankle inversion angles compared to NT, supporting previous findings. Prior research has shown that excessive inversion of the ankle leads to increased medial displacement of the talus, which in turn heightens tension in the lateral ankle ligaments, a primary mechanism for ankle sprains [51,53]. Ankle joint eversion, as the opposite movement to inversion, can counteract excessive ankle inversion during landing and prevent the transfer of load to the lateral side of the ankle, thereby reducing the risk of lateral ankle sprains. The results of this study indicate that KT significantly decreases the inversion angle of the ankle during the landing phase; thus, using KT during exercise may lower the risk of injury. Sarvestan et al. [24] employed a single-leg hopping maneuver to assess patients with CAI. KT plays a significant role in improving the movement patterns of the ankle, knee, and hip joints, with a particular focus on reducing inversion angles, enhancing knee flexion, and optimizing hip flexion. These modifications help mitigate the transmission of GRF to the lower-limb joints, thereby decreasing the risk of injury. In rehabilitation settings, KT can serve as an adjunctive tool to support the maintenance of correct movement patterns during the recovery process. By adjusting the kinematic features of the ankle, knee, and hip, KT facilitates the gradual development of more stable movement patterns, ultimately lowering the risk of injuries associated with improper loading or movement patterns. The results indicated a significant reduction in the angular velocity of the ankle joint in the sagittal plane following the application of KT. This finding suggests a slower loading speed during sagittal plane motion of the ankle joint. During the single-leg landing process, the observed decrease in angular velocity may imply an improvement in ankle joint control, potentially reducing the risk of lower-limb injuries associated with excessive speed [21]. Furthermore, the use of KT significantly reduced the angular velocity of the ankle joint, which may optimize its movement pattern and suggests a reduction in the incidence of ankle joint injuries. However, this study also revealed that the use of the ST increased the angular velocity of the knee, while the LT increased the angular velocity of the hip. These differences were relatively small, possibly indicating a placebo effect or suggesting that the impact may not have reached a significant level [54].

Joint moments reflect the movement trends of joints, represent the control exerted by muscles on these joints, and play a crucial role in maintaining joint stability. They serve as an effective mechanism for the human body to sustain balance during movement. Studies have demonstrated that, under identical conditions, greater force correlates with a higher probability of joint injury [55]. Furthermore, it is inappropriate to consider the moment of a single joint in isolation; rather, the kinetic chain should be viewed holistically, acknowledging its role in the comprehensive synergistic action during movement tasks. In the side-step cutting action, individuals with CAI exhibited greater knee eversion and internal rotation moments, which increased the load on the knee joint and elevated the risk of knee injury. This experimental study revealed that, under the KT application, the external moment at the knee during initial contact significantly increased compared to the NT condition, while the internal rotation moment significantly decreased. This may be attributed to the KT intervention increasing the knee inversion angle, thereby elevating the knee eversion moment, or it could be due to the negligible placebo effect of the KT length on CAI. The findings indicate that an increase in the hip flexion moment can provide enhanced buffering for adjacent joints, thereby aiding in the maintenance of hip stability and muscle strength balance. In this study, following the application of ST, the hip flexion moment exhibited a tendency to increase when compared to the NT, although no significant difference was observed with the LT. The hip serves as a critical source of support in the coronal plane. This experimental study revealed that under LT application, the hip abduction moment increased in comparison to ST, suggesting that KT application may enhance the hip abduction moment at initial contact. Additionally, a large knee eversion moment has been recognized as a risk factor for predicting injury and may contribute to the injury mechanism [56]. Researcher Sigward found that when subjects performed lateral cutting movements with a larger knee eversion angle, their knee eversion moment increased sixfold compared to those with a normal eversion angle. This indicates that subjects with a larger knee eversion angle adopted a different contact pattern during lateral cutting movements, resulting in increased lateral ground reaction forces and, consequently, a greater knee eversion moment [57]. Further research could explore this phenomenon in more detail. Studies indicate that a decrease in ankle plantar flexion and inversion moments is crucial for reducing ankle load. In this study, the use of LT significantly reduced the ankle inversion moment, and this biomechanical change aids in restoring normal joint load rates, preventing ankle degeneration, and is also highly significant in reducing the risk of ankle injury. Individuals with CAI exhibit greater anterior shear forces in the ankle during both the early and late stance phases compared to those without CAI [30,58]. The application of KT has been shown to reduce the ankle joint reaction force. This therapeutic intervention, which involves the strategic placement of adhesive tape on the skin, enhances muscle activation and provides additional support to the joint, thereby mitigating the forces acting upon it. These findings suggest that KT may improve proprioception, alter muscle tone, and enhance joint stability, all of which contribute to the overall reduction in joint loading. KT can be a crucial component of a comprehensive rehabilitation program, alongside other therapeutic interventions such as strength training, proprioceptive exercises, and mobility work. Clinicians can utilize KT to enhance joint stability and mobility, tailored to the patient’s specific injury history, movement patterns, and recovery progress.

This study has several limitations. Firstly, considering the prevalence of CAI and LAS, a notable limitation of this study is its small sample size, which may restrict the generalizability of the results. As such, future studies should aim to expand the sample size. Secondly, all lateral cutting movements were performed under anticipated conditions, without simulating real-life scenarios, as most side-step cutting movements occur under unanticipated conditions. Therefore, further analysis is warranted to compare anticipated and unanticipated conditions. Moreover, the KT application method employed in this study exclusively involved joint taping. Future investigations could benefit from exploring various taping methods or comparing the effects of taping on different anatomical regions to determine which method most effectively protects athletes during side-step cutting movements. Lastly, while this study provided preliminary insights into the kinematic and kinetic effects of KT on CAI, it did not investigate the underlying mechanisms. Future studies should seek to collect a broader range of data, including participants’ subjective feedback and electromyography, to elucidate the mechanisms by which KT exerts its effects.

## 5. Conclusions

In conclusion, the application of LT significantly decreased the inversion moment and joint reaction force at the ankle joint, which may enhance shock absorption upon foot contact, thereby reducing the impact of ground reaction forces. These interventions could potentially aid in the prevention of lower-limb injuries in patients with CAI. Further studies are necessary to explore the long-term benefits of these taping methods and their incorporation into comprehensive injury prevention programs for athletes and individuals with CAI.

## Data Availability

The Ethics Committee of Ningbo University reviewed and approved this research involving human participants. Participants submitted their written informed consent to engage in this study. The authors will ensure that the raw data supporting the results of this paper are freely accessible.

## Supplementary Materials

The following supporting information can be downloaded at: https://www.mdpi.com/article/10.3390/healthcare12242561/s1. Figure S1. Group comparisons of joint angles across the stance phase of cutting. Figure S2. Group comparisons of joint angular velocities across the prelanding phase of cutting. Figure S3. Group comparisons of joint moments across the descending phase of cutting.
