# Supplementary material for: The Biomechanical Effects of Kinesiology Taping Methods on Side-Step Cutting Movements in Chronic Ankle Instability
Source: Healthcare (Basel). 2024 Dec 19;12(24):2561. doi: 10.3390/healthcare12242561 (PMC11675757; doi:10.3390/healthcare12242561)
Supplement: Supplementary file 1 [file healthcare-12-02561-s001.zip › healthcare-3321315-supplementary.pdf]

Joint angles during entire phase

As is shown in Figure S1, no significant differences were observed for those joint angles during the stance phase of cutting.

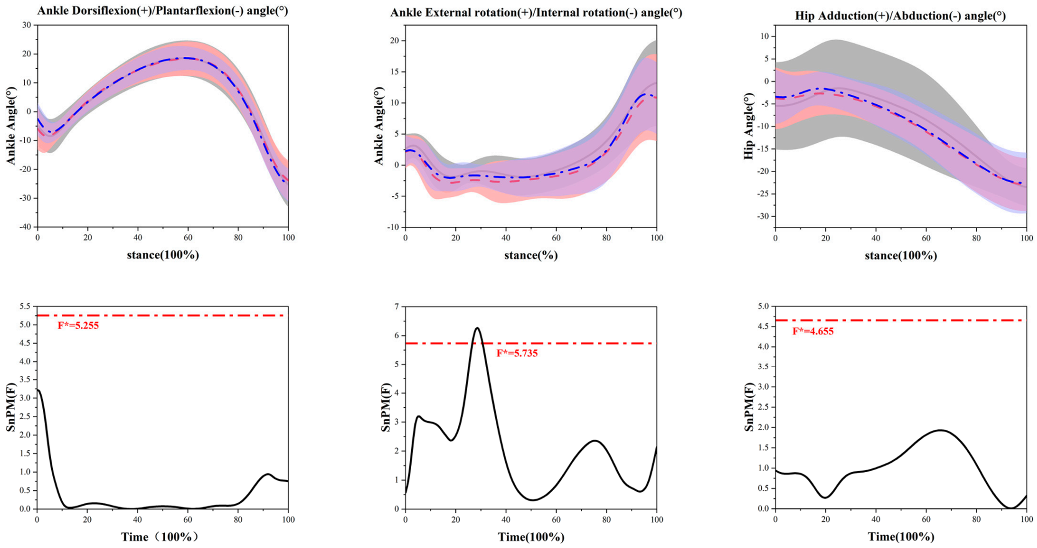

Figure S1. Group comparisons of joint angles across the stance phase of cutting.

Joint angular velocities during entire phase

As is shown in Figure S2, no significant differences were observed for those joint angular velocities during the stance phase of cutting.

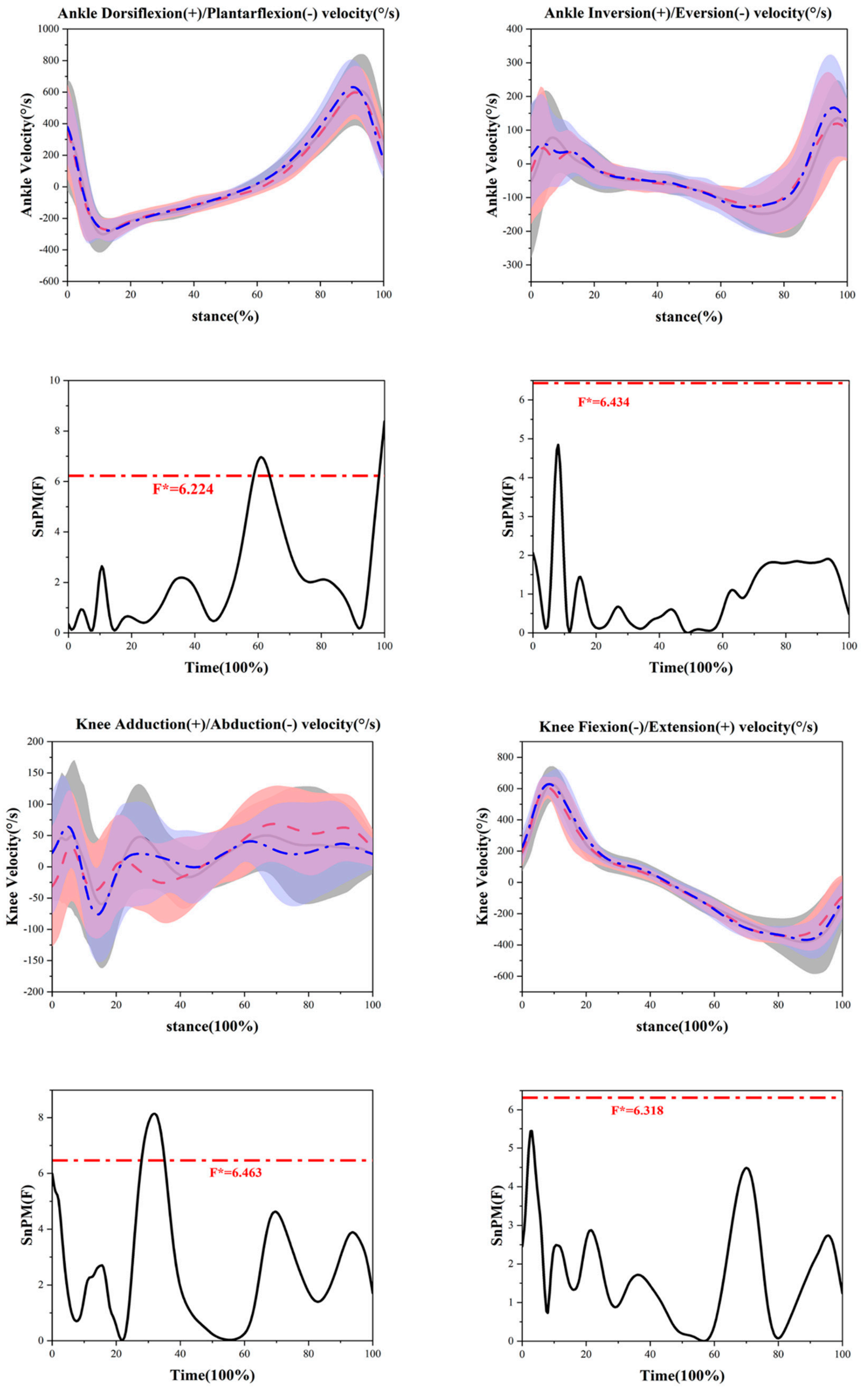

Figure S2. Group comparisons of joint angular velocities across the prelanding phase of cutting.

Joint moments during entire stance phase

In terms of the joint moments, no significant differences were observed in the cutting (Figure S3).

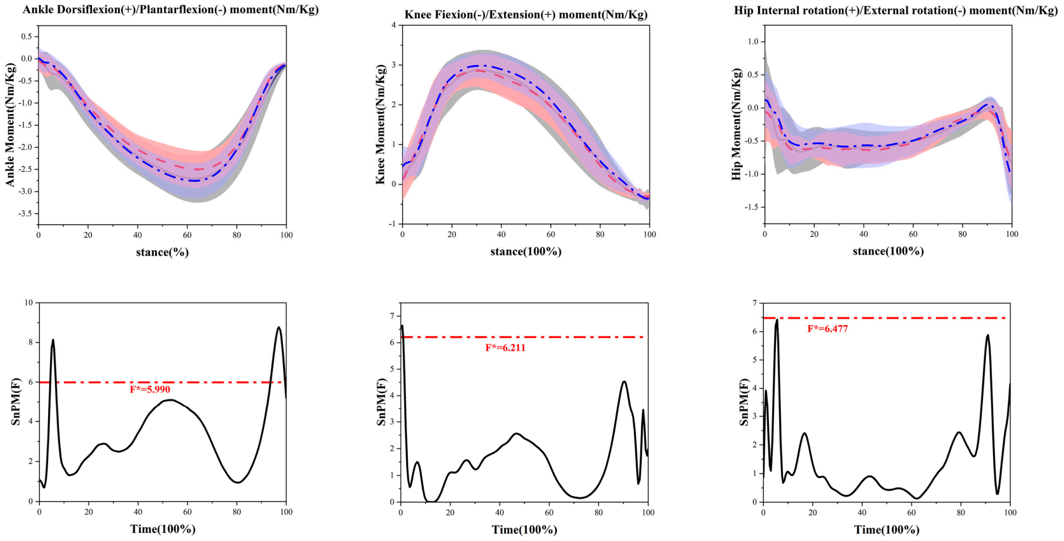

Figure S3. Group comparisons of joint moments across the descending phase of cutting.
